# Gender and medication use in Turkey: Evidence from a general population survey

**DOI:** 10.1371/journal.pone.0321590

**Published:** 2025-04-09

**Authors:** Tekin Kose

**Affiliations:** 1 School of Business and Law, University of Brighton, Brighton, The United Kingdom; 2 Department of Economics, TED University, Ankara, Turkey; Public Library of Science, UNITED KINGDOM OF GREAT BRITAIN AND NORTHERN IRELAND

## Abstract

Gender differences in health behaviors and outcomes were commonly documented by researchers. The focus of this study was the analysis of gender differences in medication use for a general population in Turkey. It also explored a range of factors associated with medication use at the individual level. A nationally representative cross-sectional data set was obtained from the 2019 wave of the Turkish Health Survey. The sample of this study included 17,083 adults residing in different regions of Turkey. Conditional mixed-process regression models were estimated for the whole sample and subsamples by gender. The rates of prescribed and non-prescribed medication use were 40.7% and 30.2%, respectively, in the adult population of Turkey. There were significant gender differences in medication use in the Turkish case. Females were 19.4% more likely to use prescribed medication, and they were 30.8% more likely to use non-prescribed medication compared to males in Turkey. There were negative associations between prescribed and non-prescribed medication use. On average, females were 9.2% less likely to report higher levels of health status, and they were 18.4% more likely to use healthcare services. Individuals with higher levels of self-rated health status were less likely to use prescribed medication. Both prescribed and non-prescribed medication use were positively related to healthcare service use. Complementing the earlier literature, the results of the present study demonstrated that gender-specific designs should be considered by health policies on the use of medications.

## Introduction

Men and women differed with respect to their health behaviors and outcomes. For individuals over the age of 15, according to Eurostat data for 2019, more than 52% of females and more than 43% of males reported that they used prescribed medication within the last two weeks in 27 European Union countries [1]. Similarly, the prevalence of non-prescribed medication use was more than 27% for males and more than 37% for females in European countries [1]. Decisions about medication use were associated with biological, social, demographic, and economic factors [2,3]. The conceptual framework for health service use was provided by Andersen (1968) and it is extended by further research [4–7]. The conceptual model framework for Andersen`s behavioral model of health service use categorized factors that impact use of health services into three major groups: 1) predisposing, 2) enabling, and 3) needs [7,8]. Predisposing factors included demographics and social factors. Enabling factors were associated with the policy, financing, and organization of health services. Needs included factors such as body mass index, physical health, and mental health status indicators that lead to actions for the use of health services. Many studies provided empirical tests of the conceptual model for various cases [6–10]. Researchers also indicated that there were multidirectional relationships among the use of health services, health behaviors, health outcomes, and factors pointed out in the model [7–8].

Empirical evidence provided diversified findings on the relationships between gender and the medication use practices of individuals [11–17]. Some researchers found that women have higher total medication use rates than men [18,19]. Additional evidence indicated that women were more likely to use prescribed medications than men [11,20–24]. There were also studies that reported no gender differences in prescribed medication use [25]. A frequent finding indicated that females were more likely to use non-prescription medication [10–12,17,23,24,26–32]. However, some researchers observed that men were more likely to utilize non-prescribed medication [19,33]. Furthermore, some studies found no significant associations between gender and non-prescribed medication use [34–39]. While the extensive literature presented valuable findings, there were limitations and gaps that need to be addressed by additional studies. The mixed findings on the associations of gender with diverse types of medication use may be attributed to multiple factors, such as heterogeneities in data collection, measurement, and analysis methods, sampling differences, and variation in health policies across countries. Many studies used small data sets collected by convenience or purposive sampling methods from local or regional populations. There were also variations in survey measurement tools, questions, and data analysis methodologies. A branch of literature employed multivariate methods, whereas some utilized bivariate analysis, and most ignored simultaneous associations across variables of interest.

Despite the valuable insights on gender differences in medication use, there was no consensus reached among the earlier studies. The primary objective of this research is to make contribution to the current literature by providing empirical analysis for the Turkish case. Turkey introduced transformation programs for health care policies and currently offers universal public health insurance, and individuals were also allowed to obtain several types of private health insurance. In this context, this research had multiple objectives and contributions. First, the author employed a large, nationally representative survey from a developing country to investigate associations between gender and medication use. Second, a specific estimation framework was utilized to analyze gender differences in medication use. Third, unlike earlier studies, the statistical methodology of this research accounted for endogeneities between health status, healthcare service utilization, and medication use. Finally, findings from the Turkish case for medication use would provide additional insights for health policy decisions, and the management of health care in developing countries.

## Materials and methods

### Data and variables

The author utilized a cross-sectional data set from the 2019 wave of the Turkish Health Survey (THS) [40]. THS 2019 was a nationally representative survey, and it was conducted by the Turkish Statistical Institute (TSI). THS 2019 used stratified sampling techniques and covered 9,470 households from various regions of Turkey. Individuals from 8,166 different households participated in the survey, and this corresponded to a response rate of 86.2% at the household level. The survey was conducted with 17,083 participants who were at least 15 years old. It collected individual-level information on demographics, socioeconomic conditions, health conditions, health-related behavior, health care access, and health service utilization. The data collection process of this study did not require ethical approval for the use of human subjects since it uses a secondary data set. The data set was provided by the TSI through official application and approval processes.

For data analysis of this study four primary outcome variables were created: 1) prescribed medication use; 2) non-prescribed medication use; 3) healthcare service use; and 4) self-rated health status. Non-responses or missing observations were excluded from the analysis by list-wise deletion. Measures for *prescribed medication use* and *non-prescribed medication use* by individuals were based on the following survey questions:

1) “During the past two weeks, have you used any medications that were prescribed for you by a doctor? 1-Yes 2-No.” If an individual reported use of any prescribed medication in the past two weeks, then the variable *prescribed medication use* was equal to one; otherwise, it was zero.
2) “During the past two weeks, have you used any medications not prescribed by a doctor? 1-Yes 2-No.” If an individual reported use of any non-prescribed medication in the past two weeks, then the variable *non-prescribed medication use* was equal to one; otherwise, it was zero.

*Healthcare service use* considered whether the respondent used the following healthcare services within the last year: a) visit a hospital; b) visit a physician; c) visit a psychologist, psychotherapist, or psychiatrist. This ordered variable ranged from zero (no service use) to three (use of all three types of service). *Self-rated health status* was based on the corresponding survey question: “How is your health in general?” This ordered variable was recorded as follows: 1-Very bad 2-Bad 3-Fair 4-Good 5-Very good.

Data analysis considered independent variables for the demographics, socioeconomic factors, and health-related information of individuals. Table 1 presented definitions and measurement details for all variables.

**Table 1:** Description of variables.

*Variable*	*Description*
Prescribed Medication Use	1 = Respondent used prescribed medication in the last 2 weeks; 0 = Respondent did not use prescribed drugs in the last 2 weeks.
Non-Prescribed Medication Use	1 = Respondent used non-prescribed medication in the last 2 weeks; 0 = Respondent did not use non-prescribed drugs in the last 2 weeks.
Self-Rated Health Status	1 = Very Bad; 2 = Bad; 3 = Fair; 4 = Good; 5 = Very Good.
Healthcare Service Use	Measures whether the respondent used following healthcare services within the last year: a) visit hospital, b) visit a physician, c) visit a psychologist, psychotherapist, or psychiatrist. 0 = Respondent did not use any healthcare service in the last year. 1 = Respondent used a healthcare service in the last year. 2 = Respondent used two types of healthcare service in the last year. 3 = Respondent used three types of healthcare service in the last year.
Female	1 = Female; 0 = Male.
Age Level	1 = 15-24; 2 = 25-34; 3 = 35-44; 4 = 45-54; 5 = 55-64; 6 = 65-74; 7 = 75 + .
Education Level	1 = No formal education or diploma; 2 = Primary school degree; 3 = Middle school degree; 4 = High school degree; 5 = College or graduate degree.
Employed	1 = Respondent is working; 0 = Otherwise.
Household Income Level	Measures monthly income level of respondent’s household in Turkish Liras (TL). 1 = Lowest income group = 0-1688 TL; 2 = Low-income group = 1669-2424 TL; 3 = Middle income group = 2425-3398 TL; 4 = Upper income group = 3399-5052 TL; 5 = Highest income group = 5053-8913 + TL.
Married	1 = Respondent is married; 0 = Otherwise.
Body Mass Index (BMI)	BMI for the respondent (kg/m^2^).
Chronic Illness	1 = Respondent has a chronic illness; 0 = Otherwise.
Health Insurance	1 = Respondent has a health insurance; 0 = Otherwise.
Smoking Level	0 = Non-Smoker; 1 = Ex-Smoker; 2 = Occasional Smoker; 3 = Daily Smoker
Alcohol Use Level	0 = Non-Drinker; 1 = Ex/Rare Drinker; 2 = Occasional Drinker; 3 = Drinker; 4 = Frequent Drinker
Vegetable Consumption Level	0 = Never; 1 = Less than once a week; 2 = 1 to 3 times a week; 3 = 4 to 6 times a week; 4 = Once or more a day
Fruit Consumption Level	0 = Never; 1 = Less than once a week; 2 = 1 to 3 times a week; 3 = 4 to 6 times a week; 4 = Once or more a day
Physical Activity Days	Number of days in a week that respondent walks for at least 10 minutes.
Household Size	Number of individuals residing in respondent’s household.

Source: TSI (2019).

### Statistical analysis

First, the author conducted a descriptive analysis for the data set of this study. Summary statistics and frequency distributions were calculated for all relevant variables. Two sample comparison tests were conducted for the proportions and means of relevant variables for females and males. Then, the author utilized an empirical analysis that accounted for endogenous relationships between the use of various healthcare services and health status. For instance, individuals may decide to use prescribed and non-prescribed medication simultaneously. An individual may consider prescribed and non-prescribed medication as substitutes and, therefore, may decide not to visit a doctor. Furthermore, the level of perceived health status may simultaneously impact decisions on medication and healthcare service use by individuals. Therefore, the empirical framework employed conditional mixed-process regression models (CMP) [41], due to the nature of the data on the health outcomes of individuals. CMP constructed a simultaneous equation system and allowed estimation of various multiple regression models with several types of variables. Additionally, CMP had the advantage of providing estimations for correlations between multiple dependent variables. Since medication use choices, health status, and healthcare service use practices of individuals were not independently determined, the author estimated a simultaneous equation framework where prescribed medication use (PMU_i_), non-prescribed medication use (NPMU_i_), self-rated health status (SRH_i_), and health service use (HSU_i_) were classified as dependent variables. The theoretical model was described by the following system of equations:


$$PMU_{i}=P_{i}\beta +e_{i}$$ (1)

$$NPMU_{i}=Q_{i}\alpha +u_{i}$$ (2)

$$SRH_{i}=R_{i}\theta +v_{i}$$ (3)

$$HSU_{i}=S_{i}\lambda +w$$ (4)

P_i_, Q_i_, R_i_, and S_i_ represented the vectors of independent variables for each equation, respectively. The vectors of parameters were denoted by ${\text{β=(β}}_{\text{1}}{\text{,β}}_{\text{2}}\text{,…}..{\text{,β}}_{\text{j}}\text{)}$, ${\text{α=(α}}_{\text{1}}{\text{,α}}_{\text{2}}\text{,…}..{\text{,α}}_{\text{k}}\text{)}$, ${\text{θ=(θ}}_{\text{1}}{\text{,θ}}_{\text{2}}\text{,…}..{\text{,θ}}_{\text{m}}\text{)}$ and ${\text{λ=(λ}}_{\text{1}}{\text{,λ}}_{\text{2}}\text{,…}..{\text{,λ}}_{\text{r}}\text{)}$. Disturbance terms of equations (e_i_, u_i_, v_i_, and w_i_) were assumed to be normally distributed with pair-wise correlations, *ρ*_st_. Given the binary nature of PMU_i_ and NPMU_i_, their corresponding regression models were estimated by the standard probit framework. Since SRH_i_ and HSU_i_ were ordered response variables, corresponding regressions were estimated by the ordered probit approach. The simultaneous equation system and correlation coefficients of error terms were estimated by employing the CMP procedures of Rodman (2011) in STATA 17 [41,42]. The systems of equations were estimated for the full sample, the female sample, and the male sample.

## Results

### Descriptive findings

Summary statistics of relevant variables for the full sample were displayed in Table 2. The total sample included 54.4% females. Overall, 40.7% of participants reported using prescribed medication, and 30.2% used non-prescribed medication. The average self-rated health status was 3.53 (0.81) for the total sample, and for males, their scores (3.66, 0.78) were higher, significantly different from females (3.42, 0.82) on average at the 1% significance level.

**Table 2:** Descriptive statistics.

*Variable*	*N*	*Mean*	*Standard Deviation*	*Min*	*Max*
Prescribed Medication Use	17,083	0.407	0.491	0	1
Non-Prescribed Medication Use	17,083	0.302	0.459	0	1
Self-Rated Health Status	17,083	3.534	0.817	1	5
Healthcare Service Use	17,083	1.264	0.836	0	3
Female	17,083	0.544	0.498	0	1
Age Level	17,083	3.447	1.740	1	7
Education Level	17,083	2.963	1.322	1	5
Employed	17,083	0.382	0.486	0	1
Household Income Level	17,083	3.073	1.363	1	5
Married	17,083	0.686	0.464	0	1
Body Mass Index (BMI)	17,083	26.48	5.070	13.42	57.28
Chronic Illness	17,083	0.625	0.484	0	1
Health Insurance	17,083	0.927	0.260	0	1
Smoking Level	17,083	1.036	1.289	0	3
Alcohol Use Level	17,083	0.456	0.858	0	4
Vegetable Consumption Level	17,083	3.290	0.932	0	4
Fruit Consumption Level	17,083	3.000	1.101	0	4
Physical Activity Days	17,083	4.556	2.763	0	7
Household Size	17,083	3.337	1.610	1	17

Source: TSI (2019).

The mean of healthcare service use was 1.26, indicating that participants used more than one service on average. The mean age of participants fell into the range of 35-44 years. The average level of education was at the level of middle school. The employment rate of the sample was 38.2% and the average household income level corresponded to middle income group. Married individuals constituted 68.8% of the sample, and the average household size was more than three people. The average body mass index was 26.48 (5.07) for the total sample, and for females, their scores (26.67, 5.59) were higher, significantly different from males (26.26, 4.36) on average at the 1% significance level. More than 62% of participants reported chronic conditions, and 92.7% of the sample had health insurance. On average, participants reported lower levels of smoking and alcohol use. Overall, the sample reported elevated levels of vegetable and fruit consumption, 4 to 6 times a week. The average days of physical activity over a week were 4.55 (2.76) for the total sample, and for males, their scores (5.10, 2.62) were higher, significantly different from females (4.09, 2.79) on average at the 1% significance level.

According to Table 3, the characteristics of female and male samples displayed significant differences. The prevalence of prescribed medication use for females (46.56%) was significantly higher than that of males (33.75%). Similarly, prevalence of non-prescribed medication use was significantly higher for females (32.96%) compared to males (26.97%). Males systematically and significantly reported higher levels of self-rated health status than females. More than 26% of men did not use any healthcare services, whereas more than 43% of females used more than one service. Females were more likely to live with chronic illnesses, have health insurance, utilize multiple healthcare services, and frequently consume fruit and vegetables. Males were more likely to be educated, employed, married, have a higher level of household income, be regular smokers, and be regular drinkers. On average, males reported a lower body mass index, higher physical activity, and living with more household members.

**Table 3:** Characteristics of participants across gender.

	Females (N = 9,300)		Males (N = 7,783)		Test Statistic
*Variables*	N	*% or Mean(sd)*	N	*% or Mean(sd)*	*|z | *
Prescribed Medication User	4,330	46.56%	2,627	33.75%	16.97^***^
Non-Prescribed Medication User	3,065	32.96%	2,099	26.97%	8.49^***^
Self-Rated Health Status					
Very Bad	162	1.74%	65	0.84%	0.51
Bad	1,079	11.60%	575	7.39%	2.70^***^
Fair	3,195	34.35%	2,019	25.94%	6.39^***^
Good	4,368	46.97%	4,373	56.19%	8.62^***^
Very Good	496	5.33%	751	9.65%	2.76^***^
Healthcare Service Use					
No Service Use	1,559	16.76%	2,052	26.37%	6.88^***^
Used One Type of Healthcare Service	3,220	34.62%	2,811	36.12%	1.22
Used Two Types of Healthcare Services	4,032	43.35%	2,717	34.91%	6.94^***^
Used Three Types of Healthcare Services	489	5.26%	203	2.61%	1.53
Age Level					
15-24	1,425	15.32%	1,305	16.77%	1.03
25-34	1,704	18.32%	1,366	17.55%	0.55
35-44	1,842	19.81%	1,553	19.95%	0.10
45-54	1,616	17.38%	1,302	16.73%	0.46
55-64	1,355	14.57%	1,158	14.88%	0.22
65-74	861	9.26%	728	9.35%	0.06
75 +	497	5.34%	371	4.77%	0.38
Education Level					
Illiterate or No Diploma	1,802	19.38%	392	5.04%	6.88^***^
Primary School	3,104	33.38%	2,508	32.22%	0.92
Middle School	1,370	14.73%	1,595	20.49%	4.09^***^
High School	1,535	16.51%	1,710	21.97%	3.92^***^
College or Graduate Degree	1,489	16.01%	1,578	20.27%	3.05^***^
Employed	2,050	22.04%	4,477	57.52%	47.53^***^
Household Income					
Lowest	1,708	18.37%	1,018	13.08%	3.61^***^
Low	2,108	22.67%	1,749	22.47%	0.15
Middle	1,721	18.51%	1,469	18.87%	0.26
Upper	2,109	22.68%	1,951	25.07%	1.79*
Highest	1,654	17.78%	1,596	20.51%	1.98^**^
Married	6,278	67.51%	5,447	69.99%	3.48^***^
Body Mass Index (BMI)	9,300	*26.67 (5.59)*	7,783	*26.26 (4.36)*	*5.23* ^***^
Has Chronic Illness	6,440	69.25%	4,244	54.53%	19.79^***^
Has Health Insurance	8,743	94.01%	7,094	91.15%	7.17^***^
Smoking Frequency					
Non-Smoker	6,761	72.70%	2,494	32.04%	35.71^***^
Ex-Smoker	739	7.95%	1,858	23.87%	9.27^***^
Occasional Smoker	314	3.38%	271	3.48%	0.07
Daily Smoker	1,486	15.98%	3,160	40.60%	16.68^***^
Alcohol Use Frequency					
Non-Drinker	8,169	87.84%	4,415	56.73%	39.53^***^
Ex/Rare Drinker	420	4.52%	1,532	19.68%	7.43^***^
Occasional Drinker	604	6.49%	1,303	16.74%	6.09^***^
Drinker	100	1.08%	426	5.47%	1.88*
Frequent Drinker	7	0.08%	107	1.37%	0.29
Vegetable Consumption					
Never	41	0.44%	58	0.75%	0.19
Less than once a week	276	2.97%	301	3.87%	0.59
1 to 3 times a week	1651	17.75%	1,683	21.62%	2.81^***^
4 to 6 times a week	1775	19.09%	1,551	19.93%	0.61
Once or more a day	5557	59.75%	4,190	53.84%	5.83^***^
Fruit Consumption					
Never	205	2.20%	137	1.76%	0.28
Less than once a week	674	7.25%	587	7.54%	0.20
1 to 3 times a week	2,371	25.49%	2,155	27.69%	0.95
4 to 6 times a week	1,462	15.72%	1,405	18.05%	1.67^*^
Once or more a day	4,588	49.33%	3,499	44.96%	3.90^***^
Physical Activity Days	9,300	*4.09 (2.79)*	7,783	*5.10 (2.62)*	*24.01* ^***^
Household Size	9,300	*3.29 (1.63)*	7,783	*3.39 (1.58)*	*3.87* ^***^

Source: TSI (2019). Notes: *sd*=standard deviation. *z* = z-values from two sample equality tests for means and proportions.

*** p < 0.01,

** p < 0.05,

* p < 0.1.

### Findings for associations of gender, medication use, healthcare service use, and health status

Findings from CMP models are displayed in Table 4 for the full sample, Table 5 for females, and Table 6 for males. Robust standard errors are utilized for all regression models. According to Table 4, there are significant gender differences in medication use, self-rated health status, and healthcare service use in Turkey. Females are 19.4% (OR =  1.194 [95% CI: 1.137, 1.254]) more likely to use prescribed medication, and they are 30.8% (OR =  1.308 [95% CI: 1.247, 1.373]) more likely to use non-prescribed medication. On average, females are 9.2% (OR =  0.908 [95% CI: 0.871, 0.947]) less likely to report higher levels of health status, and they are 18.4% (OR =  1.184 [95% CI: 1.138, 1.231]) more likely to use healthcare services.

**Table 4:** CMP estimation results: Odds ratios for full sample.

Variables	*(1) Prescribed Medication Use*	*(2) Non-Prescribed Medication Use*	*(3) Self-Rated Health Status*	*(4) Healthcare Service Use*
Female	1.194^***^	1.308^***^	0.908^***^	1.184^***^
	(1.137, 1.254)	(1.247, 1.373)	(0.871, 0.947)	(1.138, 1.231)
Age Level	1.192^***^	0.959^***^	0.819^***^	1.016^**^
	(1.173, 1.212)	(0.944, 0.975)	(0.807, 0.831)	(1.003, 1.030)
Education Level	1.000	1.009	1.113^***^	1.043^***^
	(0.980, 1.020)	(0.990, 1.029)	(1.094, 1.132)	(1.026 - 1.060)
Employed	0.848^***^	1.133^***^	1.094^***^	0.847^***^
	(0.808, 0.891)	(1.081, 1.189)	(1.050, 1.141)	(0.814, 0.881)
Household Income Level	1.013	0.991	1.083^***^	1.003
	(0.995, 1.030)	(0.974, 1.008)	(1.067, 1.099)	(0.989, 1.017)
Married	0.973	1.152^***^	1.038 *	1.056^***^
	(0.927, 1.023)	(1.099, 1.207)	(0.996, 1.081)	(1.016, 1.097)
Body Mass Index (BMI)	1.015^***^	1.007^***^	0.990^***^	1.007^***^
	(1.011, 1.020)	(1.002, 1.011)	(0.986, 0.994)	(1.004, 1.011)
Chronic Illness	2.314^***^	1.301^***^	0.241^***^	1.748^***^
	(2.205, 2.429)	(1.241, 1.363)	(0.229, 0.253)	(1.682, 1.817)
Health Insurance	1.268^***^	1.059	0.979	1.402^***^
	(1.160, 1.386)	(0.978, 1.146)	(0.911, 1.052)	(1.312, 1.498)
Smoking Level	0.970^***^	1.055^***^	0.986 *	1.011
	(0.953, 0.988)	(1.036, 1.073)	(0.971, 1.002)	(0.996, 1.026)
Alcohol Use Level	1.039^***^	1.067^***^	1.061^***^	0.974^**^
	(1.011, 1.067)	(1.040, 1.095)	(1.036, 1.086)	(0.953, 0.996)
Vegetable Consumption Level	1.028^**^	1.021 *	1.062^***^	0.951^***^
	(1.002, 1.055)	(0.996, 1.047)	(1.039, 1.085)	(0.931, 0.970)
Fruit Consumption Level	0.998	0.968^***^	1.051^***^	0.997
	(0.977, 1.020)	(0.948, 0.989)	(1.032, 1.071)	(0.980, 1.015)
Physical Activity Days	0.987^***^	1.001	1.040^***^	0.979^***^
	(0.980, 0.995)	(0.993, 1.008)	(1.033, 1.047)	(0.973, 0.986)
Household Size	0.966^***^	0.996	0.979^***^	0.990 *
	(0.951, 0.981)	(0.982, 1.010)	(0.967, 0.991)	(0.979, 1.002)
R^2^/Pseudo R^2^ from individual model	0.188	0.021	0.236	0.046
Atanhrho with prescribed medication use		-0.048^***^	-0.214^***^	0.502^***^
		(-0.075, -0.021)	(-0.238, -0.189)	(0.477, 0.525)
Atanhrho with non-prescribed medication use			-0.0158	0.0260^**^
			(-0.038, 0.007)	(0.004, 0.047)
Atanhrho with self-rated health status				-0.204^***^
				(-0.223, -0.184)
Wald χ^2^	10631.21^***^			
Number of Observations	17,083			

Source: TSI (2019). Notes:

*** p < 0.01,

** p < 0.05,

* p < 0.1. 95% confidence intervals are in parentheses.

**Table 5:** CMP estimation results: Odds ratios for female sample.

Variables	*(1) Prescribed Medication Use*	*(2) Non-Prescribed Medication Use*	*(3) Self-Rated Health Status*	*(4) Healthcare Service Use*
Age Level	1.173^***^	0.957^***^	0.818^***^	0.999
	(1.148, 1.200)	(0.937, 0.979)	(0.801, 0.835)	(0.981, 1.017)
Education Level	0.983	0.996	1.136^***^	1.027^**^
	(0.956, 1.010)	(0.970, 1.024)	(1.109, 1.164)	(1.004, 1.050)
Employed	0.902^***^	1.158^***^	1.045	0.896^***^
	(0.841, 0.969)	(1.081, 1.240)	(0.983, 1.110)	(0.846, 0.949)
Household Income Level	1.023 *	0.996	1.079^***^	1.008
	(1.000, 1.047)	(0.974 - 1.019)	(1.057 - 1.101)	(0.989 - 1.027)
Married	0.948 *	1.132^***^	1.032	1.037
	(0.891, 1.009)	(1.067, 1.202)	(0.980, 1.087)	(0.987, 1.090)
Body Mass Index (BMI)	1.020^***^	1.006^**^	0.989^***^	1.012^***^
	(1.014, 1.025)	(1.000, 1.011)	(0.985, 0.994)	(1.007, 1.016)
Chronic Illness	2.190^***^	1.346^***^	0.240^***^	1.658^***^
	(2.047, 2.344)	(1.260, 1.438)	(0.223, 0.258)	(1.570, 1.751)
Health Insurance	1.244^***^	0.985	0.980	1.374^***^
	(1.100, 1.407)	(0.880, 1.103)	(0.879, 1.092)	(1.248, 1.513)
Smoking Level	0.988	1.071^***^	0.978 *	1.042^***^
	(0.963, 1.014)	(1.045, 1.098)	(0.956, 1.001)	(1.020, 1.065)
Alcohol Use Level	1.050 *	1.094^***^	1.061^***^	0.990
	(1.000, 1.103)	(1.043, 1.148)	(1.015, 1.109)	(0.949, 1.034)
Vegetable Consumption Level	1.040^**^	1.028	1.054^***^	0.931^***^
	(1.005, 1.077)	(0.994, 1.064)	(1.023, 1.087)	(0.904, 0.958)
Fruit Consumption Level	0.993	0.966^**^	1.046^***^	0.993
	(0.965, 1.021)	(0.940, 0.993)	(1.021, 1.072)	(0.970, 1.017)
Physical Activity Days	0.984^***^	1.005	1.050^***^	0.974^***^
	(0.974, 0.994)	(0.995, 1.015)	(1.041, 1.059)	(0.966, 0.982)
Household Size	0.952^***^	1.003	0.984 *	0.984^**^
	(0.933, 0.971)	(0.984, 1.022)	(0.967, 1.001)	(0.969, 0.999)
R^2^/Pseudo R^2^ from individual model	0.164	0.0205	0.236	0.032
Atanhrho with prescribed medication use		-0.059^***^	-0.196^***^	0.479^***^
		(-0.094, -0.023)	(-0.228, -0.164)	(0.447, 0.510)
Atanhrho with non-prescribed medication use			-0.015	0.012
			(-0.046, 0.014)	(-0.016, 0.041)
Atanhrho with self-rated health status				-0.189^***^
				(-0.216, - 0.163)
Wald χ^2^	5499.94^***^			
Number of Observations	9,300			

Source: TSI (2019). Notes:

*** p < 0.01,

** p < 0.05,

* p < 0.1. 95% confidence intervals are in parentheses.

**Table 6:** CMP estimation results: Odds ratios for male sample.

Variables	*(1) Prescribed Medication Use*	*(2) Non-Prescribed Medication Use*	*(3) Self-Rated Health Status*	*(4) Healthcare Service Use*
Age Level	1.203^***^	0.954^***^	0.823^***^	1.029^***^
	(1.171, 1.235)	(0.929, 0.980)	(0.804, 0.842)	(1.008, 1.051)
Education Level	1.007	1.013	1.087^***^	1.049^***^
	(0.977, 1.038)	(0.983, 1.043)	(1.060, 1.116)	(1.024, 1.075)
Employed	0.811^***^	1.099^***^	1.129^***^	0.827^***^
	(0.754, 0.872)	(1.024, 1.180)	(1.062, 1.199)	(0.781, 0.877)
Household Income Level	0.999	0.982	1.091^***^	0.996
	(0.973, 1.026)	(0.958, 1.008)	(1.067, 1.116)	(0.976, 1.017)
Married	1.003	1.187^***^	1.035	1.062 *
	(0.917, 1.098)	(1.089, 1.295)	(0.959, 1.118)	(0.991, 1.137)
Body Mass Index (BMI)	1.011^***^	1.010^**^	0.991^***^	1.003
	(1.003, 1.019)	(1.002, 1.017)	(0.985, 0.998)	(0.997, 1.009)
Chronic Illness	2.425^***^	1.244^***^	0.243^***^	1.825^***^
	(2.264, 2.598)	(1.164, 1.330)	(0.227, 0.261)	(1.728, 1.928)
Health Insurance	1.287^***^	1.129^**^	0.969	1.418^***^
	(1.130, 1.465)	(1.009, 1.264)	(0.880, 1.068)	(1.293, 1.554)
Smoking Level	0.952^***^	1.037^***^	0.990	0.985
	(0.927, 0.977)	(1.011, 1.063)	(0.969, 1.011)	(0.965, 1.005)
Alcohol Use Level	1.041^**^	1.058^***^	1.057^***^	0.971^**^
	(1.007, 1.076)	(1.026, 1.092)	(1.029, 1.087)	(0.946, 0.998)
Vegetable Consumption Level	1.009	1.012	1.068^***^	0.970^**^
	(0.971, 1.049)	(0.976, 1.049)	(1.035, 1.102)	(0.942, 0.999)
Fruit Consumption Level	1.007	0.974	1.058^***^	1.007
	(0.974, 1.042)	(0.943, 1.006)	(1.029, 1.087)	(0.981, 1.034)
Physical Activity Days	0.990 *	0.994	1.027^***^	0.985^***^
	(0.978, 1.002)	(0.982, 1.005)	(1.017, 1.038)	(0.975, 0.994)
Household Size	0.985	0.986	0.972^***^	0.997
	(0.962, 1.008)	(0.965, 1.008)	(0.955, 0.990)	(0.980, 1.015)
R^2^/Pseudo R^2^ from individual model	0.199	0.016	0.223	0.048
Atanhrho with prescribed medication use		-0.036^*^	-0.235^***^	0.528^***^
		(-0.078, 0.006)	(-0.272, - 0.197)	(0.491, 0.564)
Atanhrho with non-prescribed medication use			-0.0164	0.0428^**^
			(-0.051, 0.018)	(0.009, 0.076)
Atanhrho with self-rated health status				-0.221^***^
				(-0.250, -0.191)
Wald χ^2^	4,631.35^***^			
Number of Observations	7,783			

Source: TSI (2019). Notes:

*** p < 0.01,

** p < 0.05,

* p < 0.1. 95% confidence intervals are in parentheses.

Estimation results indicate that there are endogenous associations among medication use, healthcare service use, and health status in Turkey. First, there are significantly negative associations between prescribed and non-prescribed medication use (atanhrhos: -0.048 [95% CI: -0.075, -0.021] for the full sample; -0.059 [95% CI: -0.094, -0.023] for females; -0.036 [95% CI: -0.078, 0.006] for males). Prescribed medication users are less likely to use non-prescribed medication, and vice versa. Second, prescribed medication use and self-rated health status display significantly negative correlations (atanhrhos: -0.214 [95% CI: -0.238, -0.189] for the full sample; -0.196 [95% CI: -0.228, -0.164] for females; -0.235 [95% CI: -0.272, -0.197] for males). Namely, individuals with higher levels of self-rated health status are less likely to use prescribed medication, and prescribed medication users are less likely to report higher levels of health. Third, prescribed medication use is positively associated with healthcare service use (atanhrhos: 0.502 [95% CI: 0.477, 0.525] for the full sample; 0.479 [95% CI: 0.447, 0.510] for females; 0.0528 [95% CI: 0.491, 0.564] for males). Individuals who utilize healthcare services are more likely to use prescribed medication, and prescribed medication users are more likely to employ healthcare services. Fourth, non-prescribed medication use and self-rated health status are not significantly related in all samples. Non-prescribed medication use is positively related to healthcare service use for the full sample (atanhrhos: 0.0260 [95% CI: 0.004, 0.047]) and males (atanhrhos: 0.0428 [95% CI: 0.009, 0.076]). Individuals who use healthcare services are more likely to utilize non-prescribed medication, and non-prescribed medication users are more likely to employ healthcare services. There are no significant associations between non-prescribed medication use and healthcare service utilization in the female sample. Finally, healthcare service use and self-rated health status display significantly negative associations (atanhrhos: -0.204 [95% CI: -0.223, -0.184] for the full sample; -0.189 [95% CI: -0.216, -0.163] for females; -0.221 [95% CI: -0.250, -0.191] for males). Specifically, individuals with higher levels of self-rated health status are less likely to use healthcare services, and healthcare service users are less likely to report higher levels of health. It should be noted that these findings are potentially influenced by nature of the data set as it covers information on healthcare service use of participants for the last two weeks. Due to limited time coverage, it would be anticipated that prescribed medicine is negatively correlated with self-rated health statuses and positively correlated with healthcare service use.

### Findings for risk factors of medication use, healthcare service use, and health status

#### Age.

According to Tables 4–6, age level was positively related to prescribed medication use. Individuals who reported higher age levels were more likely to employ prescribed medication. Moreover, older individuals were less likely to use non-prescribed medication, and they were less likely to report higher levels of self-rated health. Table 5 indicated that use of healthcare services was not related to age level for females. According to Tables 4 and 6, there were negative associations between age and healthcare service use for males and the full sample. Older males utilized a smaller number of healthcare services compared to younger males.

#### Education.

Tables 4–6 demonstrated that education level was not associated with both prescribed and non-prescribed medication use for both males and females. However, it was positively associated with self-rated health status and healthcare service use in all samples. More educated individuals were more likely to report higher levels of self-rated health. Moreover, individuals with higher levels of education used more healthcare services within the previous year.

#### Employment.

Empirical findings, given in Tables 4–6, indicated that employed individuals were less likely to use prescribed medication and health care services in all samples. However, being employed was positively associated with non-prescribed medication use. Employed individuals were more inclined to utilize non-prescribed medication in all samples. According to Table 6, employed males reported higher levels of self-rated health status. However, Table 5 revealed that there were no significant associations between self-rated health levels and being employed for female participants in Turkey.

#### Household income and size.

Tables 4–6 demonstrated that household income level was not significantly associated with non-prescribed medication use or healthcare service use in all samples. According to Tables 4 and 6, prescribed medication use was not significantly associated with household income level for males and the full sample. However, according to Table 5, household income displayed positive associations with prescribed medication use in the female sample. Female respondents with higher levels of household income were more likely to utilize prescribed medication compared to those with lower levels of household income. According to Tables 4–6, there were positively significant relationships between self-rated health status and household income in all samples. Individuals with higher levels of household income reported a better self-rated health status in Turkey.

Table 4 demonstrated that household size was negatively associated with prescribed medication. Individuals living with more members within a household were less likely to utilize prescribed medication. Tables 5 and 6 indicated that although this finding was prevalent for the sample of females, there were no associations between prescribed medication use and household size for the male sample in Turkey. Empirical results from Tables 4–6 demonstrated that non-prescribed medication use was not significantly associated with household size in the Turkish case. According to Table 4, individuals residing with a higher number of members in a household were less inclined to use healthcare services. Results from Tables 5 and 6 revealed that this finding was relevant for female participants, whereas there were no significant relations between household size and the use of healthcare services for male participants in Turkey. Finally, according to Tables 4–6, self-rated health status was negatively associated with household size for all samples. Namely, participants who lived in a household with a higher number of members reported lower levels of self-rated health.

#### Marital status.

Findings, in Tables 4–6, indicated that married individuals were more likely to use non-prescribed medication compared to unmarried individuals in all samples. According to Table 5, married females displayed a lower likelihood of using prescribed medication compared to their unmarried counterparts. Results from Tables 4 and 6 did not indicate any associations between the utilization of prescribed medication and marital status for males or in the overall sample. According to Table 4, being married is significantly and positively related to self-rated health status in the full sample. Overall, married individuals reported better health status in Turkey. However, there were no relationships between marital status and self-rated health level in sub-samples of males and females. Tables 4 and 6 demonstrated that healthcare service use was positively associated with being married for males and the full sample. Namely, married males used more healthcare services compared to their unmarried counterparts in Turkey. On the other hand, Table 5 indicated that there was no significant difference between the healthcare service use levels of married and unmarried females.

#### Body mass index.

For the Turkish case, according to Tables 4–6, individuals with higher body mass indices were more likely to take prescribed and non-prescribed medication. Additionally, participants with a higher body mass index reported higher levels of self-rated health status in all samples. Table 5 revealed that body mass index was positively associated with higher levels of healthcare service use for females. Females with higher levels of body mass index used more healthcare services. However, according to Table 6, body mass index was not associated with healthcare service use levels for the sample of males in Turkey.

#### Chronic illness.

Empirical results indicated robust associations between having a chronic illness and the four primary health outcomes. Tables 4–6 indicated that individuals with a chronic illness were more likely to use prescribed and non-prescribed medication. Namely, the likelihood of using prescribed or non-prescribed medication was higher among participants who reported a chronic illness. Moreover, respondents with a chronic illness used more healthcare services in Turkey. According to Tables 4–6, having a chronic illness was negatively associated with reporting higher levels of health status. Respondents with a chronic illness were more likely to state that they had lower levels of self-rated health.

#### Health insurance.

Results from the full sample, in Table 4, indicated that having health insurance was positively associated with the use of prescribed medication. Respondents who possessed health insurance displayed a higher likelihood of using prescribed medication. Moreover, individuals with health insurance use more healthcare services in Turkey. According to Tables 4 and 5, having health insurance was not significantly associated with non-prescribed medication use for the full sample and females. However, Table 5 revealed that males with health insurance were more likely to use non-prescribed medication. Empirical findings from Tables 4–6 demonstrated that there were no significant relations between self-rated health status and having health insurance for all samples.

#### Tobacco and alcohol use.

Results from Table 4 revealed that smoking was negatively associated with prescribed medication use in the overall sample. Table 6 indicated that males who reported higher frequencies of tobacco use were less likely to use prescribed medication. There were no significant associations between smoking level and prescribed medication use in the female sample. According to Tables 4–6, smoking levels displayed positive associations with non-prescribed medication use in all samples. Individuals who reported higher frequencies of tobacco use exhibited lower probabilities of utilizing non-prescribed medication. Table 4 demonstrated that smoking was negatively associated with self-rated health status in the overall sample. According to Table 5, females who smoked more frequently reported lower levels of self-rated health. On the other hand, Table 6 revealed that there was no significant association between smoking level and self-rated health status for males. Smoking level displayed significantly positive associations with health service use for only females. According to Table 5, females with higher frequencies of tobacco consumption used more healthcare services in Turkey. However, Tables 4 and 6 indicated that there were no relations between smoking frequency and healthcare service utilization for the overall sample and males.

Empirical findings revealed that alcohol use level was positively associated with self-rated health status, prescribed medication use, and non-prescribed medication use in all samples. Results from Tables 4–6 displayed that individuals with higher frequencies of alcohol consumption were more likely to use prescribed and non-prescribed medications. Similarly, respondents who engaged in more frequent alcohol use had a higher propensity to report a better health status. Tables 4 and 6 indicated that there were negatively significant associations between alcohol use level and healthcare service utilization for males and the full sample. Table 6 revealed that males with higher frequencies of alcohol consumption used more healthcare services in Turkey. However, according to Table 5, alcohol use level did not display significant relations with health service use for females.

#### Dietary behaviors.

In the full sample results reported in Table 4, vegetable consumption level of individuals was positively related to self-rated health, prescribed medication use, and non-prescribed medication use. Individuals who consumed vegetables more frequently were more likely to utilize prescribed and non-prescribed medication. Additionally, respondents who reported higher frequencies of vegetable consumption displayed better self-rated health statuses. Table 4 also revealed that the frequency of vegetable consumption was negatively associated with healthcare service use. The higher the frequency of vegetable consumption for individuals, the lower the level of healthcare use in Turkey. According to Table 5, the vegetable consumption level of females was positively related to the likelihood of prescribed medication use. However, Table 6 indicated that there was no meaningful relationship between vegetable consumption and prescribed medication use in the male sample. According to Tables 4–6, fruit consumption level was not associated with prescribed medication use or healthcare service use in Turkey. There were significantly positive relationships between fruit consumption and the self-rated health status of individuals. Tables 4-6 revealed that individuals who consumed fruit more frequently were more likely to report higher levels of self-rated health. Results from Tables 4 and 5 demonstrated that non-prescribed medication use and fruit consumption levels were positively associated for females and the full sample. Females with higher frequencies of fruit consumption displayed higher tendencies to use non-prescribed medication. Table 6 displayed that there were no relations between fruit consumption levels and non-prescribed for males in Turkey.

#### Physical activity.

Empirical findings from Tables 4–6 indicated that physically active individuals were likely to report better health statuses, and they were less likely to use healthcare services and prescribed medication in all samples. According to Tables 4–6, respondents with a higher number of physically active days were less likely to utilize prescribed medication, and they used fewer healthcare services in Turkey. Moreover, individuals who spent more days with walking activity reported higher levels of self-rated health status. Finally, results from Tables 4–6 revealed that there were no significant relationships between physical activity level and non-prescribed medication use among respondents in the Turkish case.

## Discussion

Health behaviors and outcomes significantly differed across genders. The main contribution of this research was the provision of empirical analysis for determinants of medication use in a developing country, Turkey, from a gender perspective. Additionally, the author made use of a nationally representative data set and an estimation method that accounted for endogeneities in health outcomes, healthcare service use, and medication use practices of individuals.

The findings confirmed that medication use practices, healthcare service use, and the self-rated health statuses of individuals displayed endogenous relationships. Prescribed and non-prescribed medication use were negatively associated. Hence, residents of Turkey treated prescribed and non-prescribed medications as substitutes. More than 40% and 30% of the general population use prescribed and non-prescribed medication, respectively, in Turkey. Both prescribed and non-prescribed medication use were significantly more prevalent in the female sample. First, these findings were in line with earlier studies that reported a higher likelihood of total medication use for females [18,19]. Second, the results complemented the line of literature that suggested females were more likely to report prescribed medication use [20–24,43]. A fraction of these large gender differences in the Turkish case may be attributed to heterogeneities in the use of different drug categories, as women were more likely to use specific products such as contraceptives. Third, results provided additional evidence for the observation that non-prescribed medication use practices were more frequently observed for females in various countries [10–12,17,23,24,26–32].

In line with earlier research, empirical findings revealed both similarities and differences for determinants of medication use across genders [9,12,22]. Age, body mass index, having a chronic illness, having health insurance, and alcohol use were positively correlated with the prescribed medication use of males and females. For the female sample only, prescribed medication use displayed positive relationships with household income and vegetable consumption. Being employed and having higher levels of physical activity were negatively related to prescribed medication use for both males and females. Married females and females living with more household members were less likely to use prescribed medication. Male smokers were less likely to use prescribed medication.

Non-prescribed medication use was positively associated with being employed, being married, having a high body mass index, having a chronic illness, smoking, and alcohol use for both females and males. For males, having health insurance was positively associated with non-prescribed medication use. Age was negatively related to non-prescribed medication use for both samples. Among females, fruit consumption level was negatively associated with prescribed medication use.

The prevalence of gender differences in medication use may be associated with lower health levels in females compared to males [13]. Another source of the gender gap may be the higher morbidity rates of women [16]. Additionally, some studies pointed out that females used more medication during their reproductive ages and visited doctors more frequently than men [16,44]. Finally, a line of literature argued that females were more prone to self-care than males, and females felt less social pressure to admit that they had pain [28,45]. Supporting these conceptual frameworks, results indicated that females were more likely to report a lower health status and employ more healthcare services.

The findings of this study had implications for public health policies within Turkey’s healthcare system. The Turkish healthcare system operates on universal health insurance coverage with complementary support from the private sector. Medication use by individuals is regulated by requirements on prescriptions and the coverage of health insurance plans. First, empirical results reported that females and males displayed different patterns of medication use. Hence, health policies on regulations and subsidization of drug use should develop interventions by considering gender differences in medication use. Second, there are differences in predictors of prescribed and non-prescribed medication use in Turkey. For instance, household income level and having health insurance are factors associated with prescribed medication use, whereas they are not significantly related to non-prescribed medication use. Public policy interventions on over-the-counter medications should account for such heterogeneities. Finally, dietary choices and physical activity are associated with the medication use decisions of females in Turkey. Health intervention programs for nutrition and drug use could be jointly constructed to address health disparities in Turkey. Overall, the findings of this research suggest that health interventions to shape medication use should incorporate gender-specific approaches. Policies that aim to reduce health disparities in developing countries should not disregard significant differences across sub-groups of the population.

Finally, this study had its own specific limitations. First, the author employed self-reported survey data, which may be subject to measurement problems and reporting biases. For instance, participants may have overstated their height, physical activity level, and healthy eating habits whereas they may have under-reported their weight, alcohol use, tobacco use and consumption of unhealthy products. Moreover, respondents may have recall bias in remembering their past behavior, which would lead to inaccuracies in the data. Survey data were also prone to social desirability bias, especially in health behavior, since participants may change their responses to match their images with social norms. Second, objective measures of health-related factors were not included in the analysis due to data limitations. Survey data provided subjective measures that potentially include framing effects, cognitive limitations of participants, and cultural interpretations. Moreover, the data set was cross-sectional and was not able to capture dynamic relationships among variables of interest over time. Specifically, the data on medication use and health service use cover limited time range of two weeks. Hence, health status, healthcare service use and medication use of participants would naturally display high correlations due to this characteristic of data collection. Third, the data set of the current study did not provide information for the use of various categories of medication. Hence, the author could not explore trends and risk factors for the use specific drug categories. Fourth, although the simultaneous equation framework of CMP considered endogenous relationships among variables for health outcomes, it did not provide causality directions due to the nature of the variables in hand. Therefore, the empirical findings of this study should still be interpreted as correlations. Additionally, like many statistical analyses, the CMP models had both linearity and normality assumptions for the variables of interest. Some health-related variables may not satisfy these assumptions, restricting the reliability of the findings. Future studies should focus on analysis of dynamic and non-linear relations among healthcare service use and health status within panel data on health outcomes. Exploration of causal pathways for medication use and analysis of specific drug categories would significantly contribute to the related research.
